# X-ray standing wave characterization of the strong metal–support interaction in Co/TiO_*x*_ model catalysts

**DOI:** 10.1107/S1600576724001730

**Published:** 2024-03-31

**Authors:** Atul Tiwari, Matteo Monai, Ksenia Matveevskii, Sergey N. Yakunin, Laurens D. B. Mandemaker, Martina Tsvetanova, Melissa J. Goodwin, Marcelo D. Ackermann, Florian Meirer, Igor A. Makhotkin

**Affiliations:** a Industrial Focus Group XUV Optics, MESA+ Institute for Nanotechnology, University of Twente, Enschede, Overijssel 7522 NB, The Netherlands; b Inorganic Chemistry and Catalysis, Debye Institute for Nanomaterials Science, Institute for Sustainable and Circular Chemistry, Utrecht University, Utrecht 3584 CS, The Netherlands; c NRC, Kurchatov Institute, Moscow 123098, Russian Federation; d Faculty of Electrical Engineering, Mathematics and Computer Science (EEMCS), Nanolab (NanoLab), University of Twente, Drienerlolaan 5, Enschede, Overijssel 7522 NB, The Netherlands; Australian Centre for Neutron Scattering, ANSTO, Australia

**Keywords:** strong metal–support interactions, SMSI, X-ray standing waves, XSW, X-ray reflectivity, XRR, grazing incidence X-ray fluorescence, GIXRF, supported metal catalysts, multilayers

## Abstract

The strong metal–support interaction (SMSI) is a phenomenon observed in supported metal catalyst systems in which reducible metal oxide supports can form overlayers over the surface of active metal nanoparticles (NPs) under a hydrogen (H_2_) environment at elevated temperatures. SMSI has been shown to affect catalyst performance in many reactions by changing the type and number of active sites on the catalyst surface. Laboratory methods for the analysis of SMSI at the nanoparticle-ensemble level are lacking and mostly based on indirect evidence, such as gas chemisorption. Here, we demonstrate the possibility to detect and characterize SMSIs in Co/TiO*_x_* model catalysts using the laboratory X-ray standing wave (XSW) technique for a large ensemble of NPs at the bulk scale. We designed a thermally stable MoN*_x_*/SiN*_x_* periodic multilayer to retain XSW generation after reduction with H_2_ gas at 600°C. The model catalyst system was synthesized here by deposition of a thin TiO*_x_* layer on top of the periodic multilayer, followed by Co NP deposition via spare ablation. A partial encapsulation of Co NPs by TiO*_x_* was identified by analyzing the change in Ti atomic distribution. This novel methodological approach can be extended to observe surface restructuring of model catalysts *in situ* at high temperature (up to 1000°C) and pressure (≤3 mbar), and can also be relevant for fundamental studies in the thermal stability of membranes, as well as metallurgy.

## Abstract 2

X-ray standing waves on a thermally stable periodic multilayer have been used to evidence the partial coverage of Co nanoparticles by a titanium oxide support in a model catalyst, following high-temperature reduction. The developed approach has potential for fundamental studies in thermally activated surface processes in catalysis, membranes and metallurgy.

## 1. Introduction

Supported catalysts comprise active metal (oxide) nanoparticles (NPs) deposited on the surface of a high-surface-area metal oxide support. Such catalysts have been extensively studied because of their relevance for industrial and environmental applications (Blaser *et al.*, 2001 ▸; Ranade & Joshi, 2016 ▸; Song, 2006 ▸). It is known that supported catalysts undergo chemical and physical changes under reaction conditions and also during activation, for example during reduction, causing NP growth and restructuring, all of which affects catalytic performance. The catalyst support can play a crucial role in stabilizing NPs and changing their structure and chemical state (Du *et al.*, 2020 ▸; Galhenage *et al.*, 2013 ▸; Gonzalezdelacruz *et al.*, 2008 ▸; Howard-Fabretto *et al.*, 2021 ▸; O’Shea *et al.*, 2011 ▸; Shaikhutdinov, 2018 ▸; Wu *et al.*, 2020 ▸). An example of this is the strong metal–support interaction (SMSI) phenomenon, which was first discovered in the work of Tauster and Fung on Pt-group metals supported on TiO_2_ (Tauster & Fung, 1978 ▸; Tauster, 1987 ▸). It was observed that metal particles supported on TiO_2_ lose their capability to adsorb H_2_ and CO significantly when reduced at elevated temperatures.

Several studies have suggested two possible factors responsible for SMSI, namely electronic and geometric effects. The electronic factor involves charge transfer between the metal particles and the support layer (Stakheev *et al.*, 2001 ▸; Ho *et al.*, 2011 ▸; Minato *et al.*, 2004 ▸; Dry, 2004 ▸). X-ray photoelectron spectroscopy (XPS) has been mainly used to characterize the electronic effect by shifts in binding energy of metal signals due to charge transfer. The formation of a thin metal oxide overlayer on the surface of the metal particles after high-temperature reduction is considered to be the geometrical factor of the SMSI (Fu *et al.*, 2005 ▸; Caballero *et al.*, 2010 ▸; Hsieh *et al.*, 2017 ▸; O’Shea *et al.*, 2011 ▸; Qin *et al.*, 2009 ▸). Such an overlayer formation has been extensively explored by *in situ* high-resolution transmission electron microscopy (HRTEM) with atomic resolution. Despite the power of HRTEM to image overlayer formation and evolution, the technique has a limited field of view where only a relatively small number of NPs can be imaged at a time. This makes statistically significant analysis time consuming, and it calls for complementary techniques to study SMSI. While XPS can be used to study SMSI at the bulk scale by measuring the signal intensity ratio between the metal and the metal oxide before and after reduction, this is also affected by NP growth and geometrical effects, which make it challenging to extract quantitative information on overlayer formation. CO chemisorption has also been routinely used since the 1980s to probe metal accessibility to the gas phase as indirect evidence of overlayer formation (Vannice, 1983 ▸). However, it does not have the potential to be extended to *in situ* or *operando* conditions. Therefore, there is a need for a complementary technique to study SMSI on a larger NP ensemble (>10^3^ NPs) and potentially *in situ* under realistic reaction conditions.

X-ray standing waves (XSWs) generated using a periodic layered structure can be used to study surfaces and buried interface profiles with elemental selectivity and sub-nanometre sensitivity to the atomic distribution. Under the Bragg condition, a standing wavefield is formed inside and above the periodic structure, giving rise to nodes and anti-nodes. The strength of the field intensity is suppressed at the nodes, whereas at the anti-nodes it is enhanced. By varying the incidence angle in the vicinity of the Bragg reflection angle, the positions of the nodes and the anti-nodes ‘travel’ through the surface layers. This XSW movement modulates the X-ray fluorescence yield from atoms present in these layers. Analyzing angle-dependent X-ray fluorescence (XRF) together with X-ray reflectivity (XRR), depth-resolved element concentration information on the surface layers and periodic structures can be obtained with a spatial accuracy at the sub-nanometre length scale (Bedzyk & Libera, 2013 ▸). This method is therefore promising for studying SMSI by measuring changes in the layers deposited on the surface of the periodic structure. It can be used to analyze geometrical modifications of the surface layer even with sub-nanometre resolution.

In this work, we demonstrate the use of XSW-modulated X-ray fluorescence to study strong metal–support interactions in TiO_*x*_-supported Co nanoparticles. Co/TiO_*x*_ catalysts are used for Fischer–Tropsch synthesis, as they have high selectivity for linear hydro­carbons, good stability and low activity for the water–gas shift reaction (Jongsomjit *et al.*, 2004 ▸; Iglesia, 1997 ▸; Howard *et al.*, 1990 ▸; Chen *et al.*, 2015 ▸; Dry, 2004 ▸; O’Shea *et al.*, 2011 ▸; Hong *et al.*, 2018 ▸, 2012 ▸). SMSI has been extensively studied for this system, making it an ideal candidate for the current proof-of-concept study (Jacobs *et al.*, 2002 ▸; Zennaroa *et al.*, 2000 ▸; Hong *et al.*, 2020 ▸; Jongsomjit *et al.*, 2005 ▸; O’Shea *et al.*, 2011 ▸; Portillo-Vélez & Zanella, 2020 ▸; Shimura *et al.*, 2013 ▸). The XSW field was generated using an MoN_*x*_/SiN_*x*_ periodic multilayer. The multilayer structure was designed and optimized to obtain maximum sensitivity toward changes in the atomic distribution of the Ti atoms of the TiO_*x*_ support layer. The signature of SMSI was detected by changes in the phase of Ti fluorescence yield before and after reduction, which is explained by changes in the Ti atomic distribution.

## 2. Materials and methods

### 2.1. Sample design and preparation

Two important considerations were taken into account while designing the sample: (i) the thermal stability of the multilayer (ML) and (ii) the thicknesses of the capping and support layer. The thermal stability of the ML structure is essential, as any expansion or contraction of the bilayers within the ML structure during annealing can potentially alter the relative position of the support layer in relation to the nodes and antinodes. This positional change of the support layer during the reduction may result in a loss of sensitivity to the movement of metal atoms from the support layer or introduce artifacts into the analysis. The MoN_*x*_/SiN_*x*_ ML was chosen to generate XSWs because the MoN_*x*_/SiN_*x*_ ML can withstand high temperatures. The number of bilayers was selected to be 20 with a period of 5.8 nm. The ML structure was tested for its thermal stability at 600°C in a vacuum environment. It was found to be thermally stable at this temperature without significant changes in the bilayer period. A comparison of X-ray reflectivity (XRR) curves for the TiO_*x*_/ML sample before and after annealing at 600°C is provided in the supporting information. The surface of the ML structure was terminated with an SiN_*x*_ layer followed by TiO_*x*_ as the support layer. The role of the top SiN_*x*_ layer is to place the TiO_*x*_ layer at the most favorable position with respect to the anti-nodes and nodes of the XSW to maximize the sensitivity of Ti atoms to it.

All samples were deposited on super-polished (root-mean-square roughness σ ≃ 0.14 nm) Si coupons with size 24.5 × 24.5 mm, diced out of 100 mm-diameter Si wafers. The periodic MoN_*x*_/SiN_*x*_ ML with 20 bilayers was deposited on top of the naturally oxidized Si substrate. The thicknesses of the MoN_*x*_ and SiN_*x*_ layers were 2.5 and 3.4 nm, respectively, having a period of 5.8 nm. The deposition was done using DC magnetron sputtering of pure Mo and Si targets in a gas mixture of Ar and N. The base pressure in the system was 8.0 × 10^−8^ mbar. The Ar and N gas flows were 25 and 20 sccm, respectively. The deposition of the TiO_*x*_ layer was done by sputtering the Ti target in a partial pressure of O_2_ (O_2_ flow of 15 sccm) and Ar (flow of Ar gas 15 sccm). Depositions were performed at a power of 36.1 W for MoN_*x*_, 204.5 W for SiN_*x*_ and 234 W for TiO_*x*_. The deposition rates were 0.011, 0.038 and 0.0048 nm s^−1^ for MoN_*x*_, SiN_*x*_ and TiO_*x*_, respectively. The thickness of the top SiN_*x*_ layer was set to 2.8 nm. Uniformity of the deposition and ion treatment was achieved by rotating the holder with substrates at 60 r min^−1^ during the entire deposition process. The thicknesses of the TiO_*x*_ layer in the TiO_*x*_/ML and Co/TiO_*x*_/ML samples were observed to be 1.8 and 2.5 nm, respectively. This difference in TiO_*x*_ layer thickness does not affect the analysis accuracy because most of the conclusions are derived from the comparison of the same samples before and after reduction.

Two samples of 10 × 10 mm size were used in this study, namely TiO_*x*_/ML and Co/TiO_*x*_/ML. Both samples have identical parts of TiO_*x*_/SiN_*x*_/[MoN_*x*_/SiN_*x*_]_×20_, and the Co/TiO_*x*_/ML samples were coated with Co NPs on top. The TiO_*x*_/ML sample was used as a reference sample whereas the Co/TiO_*x*_/ML sample was used as the main sample.

Co nanoparticles (NPs) were deposited on the TiO_2_ surface of the Co/TiO_*x*_/ML sample by spark ablation (Pfeiffer *et al.*, 2014 ▸) using a VSP-G1 nanoparticle generator equipped with Co electrodes (Co rod, 99.95%, 6 mm Ø, ChemPUR) and a diffusion cell (Wondergem *et al.*, 2020 ▸). The deposition conditions were as follows: voltage 1.3 kV, current 8.7 mA, Ar gas flow 30 l min^−1^, deposition time 1 h. The NP ‘anchoring’ step was performed on samples with Co NPs. These samples were heated in a tubular oven using 10 ml min^−1^ H_2_ and 90 ml min^−1^ N_2_ at 250°C for 1 h, with a 5°C min^−1^ temperature ramp. This sample is further referred to as ‘pristine’. To induce SMSI, the Co/TiO_*x*_/ML sample was further reduced at 600°C for 1 h using the same gas flows and temperature ramp. The TiO_*x*_/ML sample was also reduced at 600°C for 1 h as a control experiment. The samples were stored under air after passivation in 5% O_2_/N_2_. These samples are referred to herein as ‘reduced’.

### 2.2. Sample characterization

The samples were first characterized by extended XRR using a Malvern PANalytical EMPYREAN X-ray diffractometer equipped with a monochromated Cu *K*α_1_ (1.54 Å) X-ray source and a beam divergence of 0.015°. The reflectivity measurement from 0.1 to 10° was completed in 9 h in four angular segments, with a step size of 0.005° and time per step of 2 s from 0.1 to 1.8°, a step size of 0.005° and time per step of 20 s from 1.8 to 3.5°, a step size of 0.005° and time per step of 30 s from 3.5 to 5.0°, and a step size of 0.01° and time per step of 30 s from 5.0 to 10°. This allowed us to resolve Kiessig fringes at higher angles by measuring for a longer time. To collect XRF data, an AMPTEK detector with 128 eV energy resolution was used. The fluorescence signal modulated by the X-ray standing wave was measured by recording fluorescence across the first Bragg peak (BP) from 0.5 to 1.2° and the second BP from 1.2 to 2.0° with acquisition times of 30 and 480 s per point, respectively. To retrieve XRF counts of Ti and Mo, the XRF spectra were analyzed using the *PyMca* program (Solé *et al.*, 2007 ▸). The XRR and XRF measurements were made in a single run without removing the sample from the sample stage.

The experimental XRR and XRF data were analyzed using the hybrid free-form approach (Kondratev *et al.*, 2022 ▸; Novikova *et al.*, 2023 ▸), enabling precise reconstruction of the electron density profile without the need for any prior assumptions about the layered structure. In the classical approach to fitting experimental XRR data, the sample is represented as a planar layered structure, with each layer having parameters such as thickness, optical constant and inter-layer (interface) thickness (Windt, 1998 ▸). In this approach, the effect of interface roughness on reflectivity data is modeled by adjusting Nevot–Croce or Debye–Waller coefficients for specular reflection. This approach to reconstructing a layered structure provides realistic information only when the optical constant profile at an interface is assumed to be an error function profile (Névot & Croce, 1980 ▸), corresponding to the normal distribution of roughness heights, which is valid only for small roughness values. Moreover, the classical approach requires a fixed interface model, making it less effective when the profile becomes more complex. In such cases, the model may need refinement or additional sublayers to accurately describe the real structure, introducing a new degree of freedom for the profile shape. Thus, it requires a more flexible and stable approach to analyze reflectivity data.

Within free-form approximation, the sample structure is represented as an array of thin sub-layers of a given chemical composition and density. The lamellae have equal thickness, the value of which is determined by the maximum angular range (*q*_max_) of the experimental data, as follows:

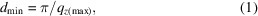

where 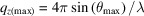 is the maximum length of the scattering vector and λ is the wavelength.

In this approach, the chemical composition of each individual sublayer is a fitting parameter. Therefore, the stoichiometry of each sublayer allows simultaneous calculation of the optical constant profile for the simulation of XRR (Yakunin *et al.*, 2014 ▸) and XSW distributions (Maderitsch *et al.*, 2018 ▸; Tiwari *et al.*, 2015 ▸) in the film for each incidence angle, and, at the same time, those atomic distribution profiles are used for the simulation of angle-dependent fluorescence yields for each chemical element (Boer *et al.*, 1995 ▸). The free form of the sample profile, with certain physical constraints, allows the reconstruction of a complex profile of extended interface, gradient and oxide layers without *a priori* knowledge of the sample structure (Zameshin *et al.*, 2016 ▸).

The analysis of XRR and XRF data results in the determination of the δ profile and the element distribution, where 1 − δ is the real part of the refractive index. It provides information about the optical constants of the material in the sample. The element distribution illustrates how each element is spatially distributed within the sample. The combined analysis helped us to reduce the cross-correlations between the model parameters during the analysis of XRR by adding an XRF dataset that is sensitive to the phase of reflected waves. This approach partially solves the well known phase problem of XRR.

Angle-resolved X-ray photoelectron spectroscopy (XPS) measurements were performed on a TiO_*x*_/ML in the pristine state. Angle-resolved XPS spectra for C 1*s* of this sample in the pristine state are given in the supporting information.

The lamella of the Co/TiO_*x*_/ML sample (after reduction at 600°C) for transmission electron microscopy characterization was prepared using a Helios 5 dual beam focused ion beam (Thermo Fisher). The surface was protected using carbon. The lamella was cut at 30 kV and then thinned alternately on the front and back sides. Polishing was performed at 5 and 2 kV. TEM characterization was conducted in a Thermo Scientific Spectra 300 transmission electron microscope with an acceleration voltage of 300 kV, a dwell time of 2.0 µs and a convergence angle of 21 mrad. The exposure time per image was 1 s. The imaging was performed with a high-angle annular dark-field detector in scanning transmission electron spectroscopy mode, with a probe current of 66 pA, a dwell time of 20 µs and a convergence angle of 21 mrad. A Super-X G2 energy-dispersive spectroscopy (EDS) detector was used to analyze the chemical composition of the films (20 µs dwell time, 100 pA beam current).

Atomic force microscopy (AFM) measurements were performed on either a pre-calibrated NT-MDT NTEGRA or a Bruker Multimode 8 system. Silicon nitride ScanAsyst-HR probes (*F* = 0.4 N m^−1^, resolution frequency = 130 kHz) were used in non-contact mode. The measurements were post-analyzed using the open-source software *Gwyddion* (https://gwyddion.net), in which a line-by-line and plain background correction were performed on a map without the particles (these were masked and excluded from the analysis). Then, the median background value and the surface roughness (expressed in RMS, including the particles) were given using the ‘Statistical functions’ function. The particles were masked using a height threshold above the background and a particle size distribution was generated, giving the distribution of the particle heights minus the median background height acquired before (this approach was applied for every individual map). For the particle size distributions shown in Fig. 3, this automated approach was not possible as the surface was too crowded and individual particles could not be automatically recognized. Therefore, the particle diameter (in the *xy* plane) was assessed manually by measuring 400 grains per image using the software’s ‘Measure distance’ function, plotted in the particle size distributions with a bin size of 10 nm.

### 2.3. Multilayer design

**Figure 1:**
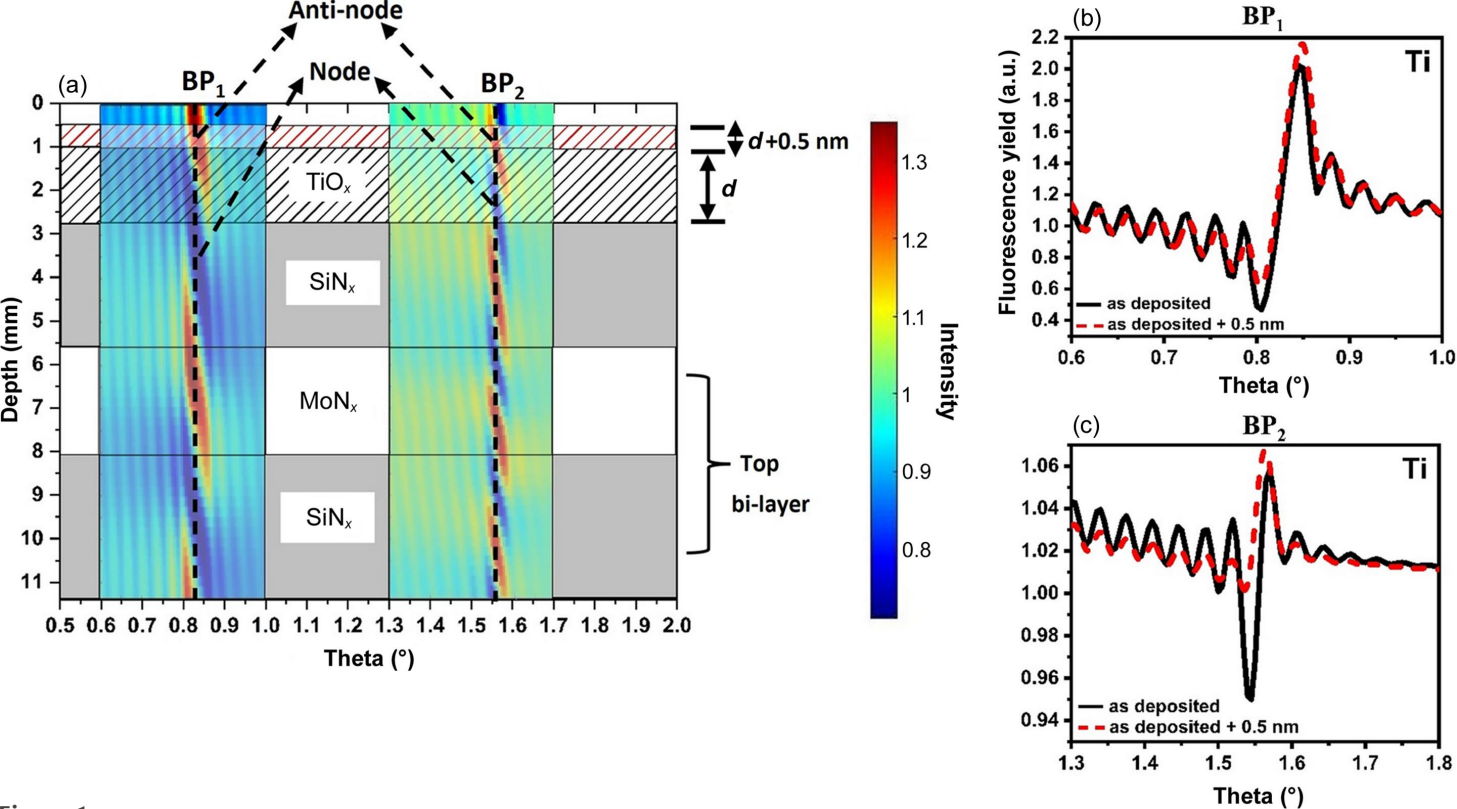
(*a*) Simulated periodic formation of nodes and anti-nodes around BP_1_ and BP_2_ inside and above the ML. For simplicity, only the top bilayer of the ML is shown. The position of TiO_*x*_, the capping layer of SiN_*x*_ and the top bilayer of the ML are shown in correlation to their overlap on nodes and anti-nodes. Ti fluorescence simulated around (*b*) BP_1_ and (*c*) BP_2_. Continuous solid lines represent the fluorescence signal of Ti from a 1.8 nm support layer, whereas the dashed lines represent the simulated Ti fluorescence signal for a layer of 2.3 nm thickness.

**Figure 2:**
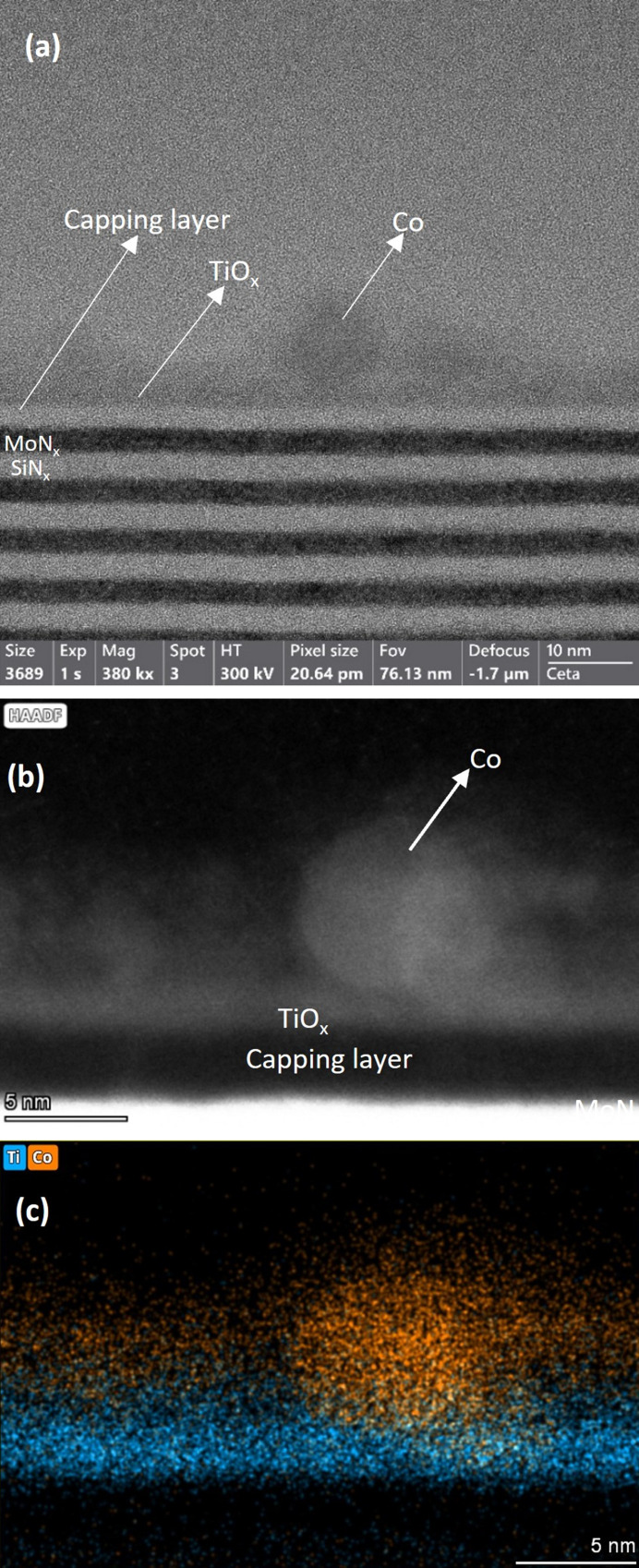
(*a*) TEM image of Co/TiO_*x*_/ML after reduction showing Co particles, TiO_*x*_ and the capping layer SiN_*x*_, and a few bilayers of the ML. (*b*) Zoomed-in dark-field image of the sample showing the Co particle, TiO_*x*_ and capping layer SiN_*x*_. (*c*) EDS chemical composition map of Co and Ti showing the distribution of Co and Ti atoms.

Fig. 1 ▸ shows the sensitivity of XSWs towards the change in Ti atomic distribution profile at the surface of an ML. Fig. 1 ▸(*a*) illustrates the periodic formation of nodes and anti-nodes of the XSW inside and above the ML around BP_1_ and BP_2_. BP_1_ and BP_2_ correspond to the maxima of the first and second BP observed in the XRR pattern, respectively. The figure shows the positions of the layers in relation to their overlap on nodes and anti-nodes. The fluorescence yield is enhanced when scanning across the anti-node and is suppressed at the node. The TiO_*x*_ support layer and SiN_*x*_ capping layer were selected to have thicknesses of 1.8 and 2.8 nm, respectively. Figs. 2 ▸(*b*) and 2 ▸(*c*) present the simulated Ti fluorescence yield around BP_1_ and BP_2_, respectively, for the TiO_*x*_ thicknesses of *d* = 1.8 and 2.3 nm (*d* + 0.5 nm). The simulation shows the sensitivity of the XSW using the best-fitting model of the TiO_*x*_/ML sample in the pristine state. In the analysis of XRR and XRF data from this sample, a low-density layer of about 1 nm was added on the TiO_*x*_ surface to create a gradual transition at the air/TiO_*x*_ interface. Notably, in Fig. 1 ▸(*a*), the outer layer starts from 0 nm and the position of TiO_*x*_ starts from 1 nm instead of 0 nm.

For a TiO_*x*_ layer thickness of *d* = 1.8 nm, the overlap of the TiO_*x*_ layer is greater on the anti-node than on the node around BP_1_, and it lies approximately between the centers of the node and the anti-node around BP_2_. The resulting Ti fluorescence yield is depicted by a continuous line in Figs. 1 ▸(*b*) and 1 ▸(*c*). When the thickness of the TiO_*x*_ layer is increased by 0.5 nm (assuming the ML is thermally stable), the position of the top interface (air or adventitious C/TiO_*x*_) is shifted upwards by the same value. Consequently, the overlap of the TiO_*x*_ layer on the node and anti-node around BP_1_ and BP_2_ changes compared with the previous position. This modified overlap of the TiO_*x*_ layer on the nodes and anti-nodes is manifested as a change in the phase of the Ti fluorescence, observed in the Ti fluorescence yield. These simulations demonstrate that even a sub-nanometre change in the atomic distribution profile, expected for Ti atoms from the TiO_*x*_ layer toward the NP surface induced by SMSI, can be detected by measuring X-ray fluorescence using standing waves.

For the correct interpretation of the results of such hybrid XRR–XRF metrology it is important to clarify the sensitivity of each individual technique, as well as their combined benefits and limitations. The XRR analysis is sensitive to electron density, meaning that it can easily resolve layers from contrast materials like Mo and Si but it is less sensitive to the difference in non-contrast materials like C and Si. Also, any layer is modeled in the approximation of lateral homogeneity, and any non-homogeneous layers (like NPs here) are modeled using the effective medium approximation. In such an approximation, the electron density profile corresponds to the electron density averaged over the normal to the substrate axis (Vorobiev *et al.*, 2015 ▸). The angle-dependent XRF measurement is sensitive to the atomic depth profiles in a thin film or to the effective media for a non-homogeneous film. In our case, we are measuring the XRF data from Ti, Mo and Co. Since the Co NPs have a variety of sizes up to ∼24 nm, which is more than twice the thickness of the period of XSW generated by the multilayer, all modulations of Co fluorescence yield will be averaged over more than one period, and therefore any reconstruction of the Co atomic distribution from Co fluorescence data will go undetected. Since the deposited Co NPs are not a continuous layer, they were modeled using effective layers for the sake of XRR fitting. The analysis of such data in the effective medium approach is beyond the scope of this paper.

## 3. Results and discussion

The synthesized Co/TiO_*x*_/ML sample was characterized using TEM and EDS techniques following reduction, to inspect the structure of the SiN_*x*_ and MoN_*x*_ multilayers and to visualize the distribution of Co and Ti atoms. Figs. 2 ▸(*a*) and 2 ▸(*b*) depict the bright-field TEM image and a magnified dark-field image of the sample, respectively. The MoN_*x*_/SiN_*x*_ ML and the capping SiN_*x*_ layer structure and composition were retained after reduction, revealing the high thermal stability of the nitride-based ML. Moreover, a ∼5 nm Co nanoparticle partially embedded in the supporting TiO_*x*_ layer was revealed by the elemental composition map of Co and Ti in Fig. 2 ▸(*c*). This is consistent with the expected encapsulation due to SMSI between Co and TiO_*x*_.

**Figure 3:**
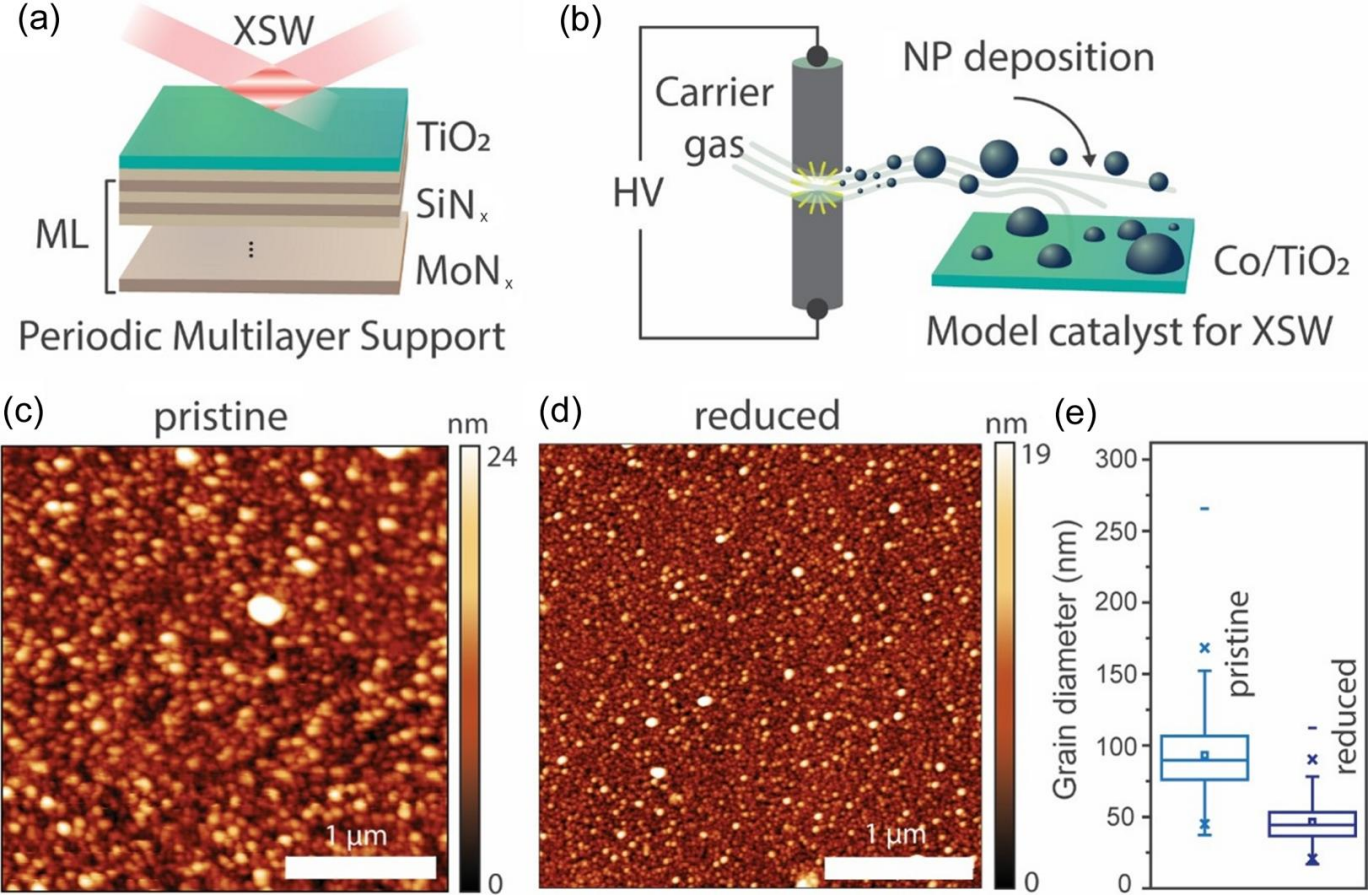
(*a*) Representation of the TiO_*x*_/ML support for XSW characterization. (*b*) Schematic of the spark ablation method for Co NP deposition on TiO_*x*_/ML. High voltage (HV) is applied to two Co electrodes in the presence of a carrier gas to induce spark discharge. The spark vaporizes some of the Co, forming an NP aerosol which is deposited on the sample by diffusion. AFM images of Co/TiO_*x*_/ML (*c*) in the pristine state and (*d*) after reduction at 600°C for 1 h. (*e*) Box charts showing the grain diameter distribution in the *xy* plane for the two model catalysts’ surfaces.

To inspect the Co NPs’ size and distribution on the TiO_*x*_ surface, the Co/TiO_*x*_/ML model catalyst prepared by spark ablation was characterized by AFM in the pristine and reduced states (Fig. 3 ▸). Although the TiO_*x*_/ML sample had a very flat surface (roughness < 0.5 nm), the Co/TiO_*x*_/ML sample in the pristine state showed islands of about 24 nm in height and 100 nm in diameter, and a surface roughness of ∼3 nm [Figs. 3 ▸(*c*) and 3 ▸(*e*)]. Such islands or grains were interpreted as the Co NPs deposited on the TiO_*x*_ surface by diffusion during spark ablation. After reduction at 600°C, the grains were about 19 nm in height, while they seemed to shrink to around 50 nm in diameter [Figs. 3 ▸(*d*) and 3 ▸(*e*)]. The decrease in grain size upon high-temperature reduction may be due to NP reconstruction and migration on the surface, and/or partial encapsulation by the TiO_*x*_ support.

**Figure 4:**
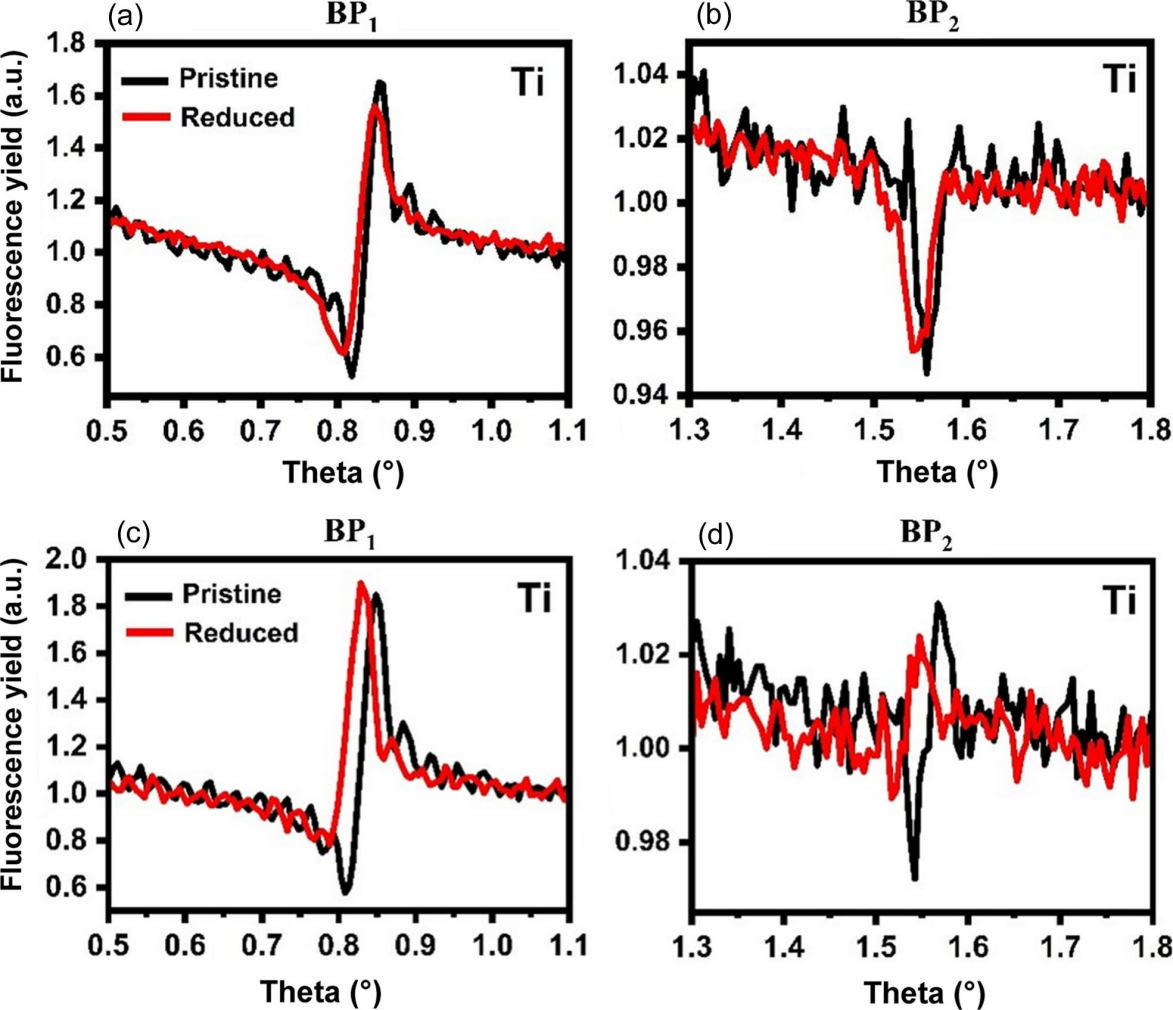
Qualitative comparison of Ti fluorescence measured around BP_1_ and BP_2_ in the pristine and reduced (*a*, *b*) TiO_*x*_/ML and (*c*, *d*) Co/TiO_*x*_/ML samples.

The Co/TiO_*x*_/ML and a reference TiO_*x*_/ML sample were analyzed by the XSW technique. Fig. 4 ▸ shows a comparison of Ti fluorescence yield measured for the TiO_*x*_/ML and Co/TiO_*x*_/ML samples in the pristine and reduced states. Figs. 4 ▸(*a*) and 4 ▸(*b*) show the Ti fluorescence yields of the TiO_*x*_/ML sample, measured around BP_1_ and BP_2_, respectively. The fluorescence yields of the sample in the pristine and reduced states almost overlap. Small curve changes can be explained by minor Ti profile modifications compared with the initial thickness of the layer.

Figs. 4 ▸(*c*) and 4 ▸(*d*) give the Ti fluorescence yield of the Co/TiO_*x*_/ML sample measured around Θ_1_ and Θ_2_, respectively. After the reduction of the Co/TiO_*x*_/ML sample, changes in the Ti fluorescence were observed. These changes can be attributed to an increase or shift of the Co/TiO_*x*_ interface towards the surface. This shows that the Ti atomic rearrangement in the TiO_*x*_ layer can be noticed qualitatively even without data fitting. A detailed explanation of the qualitative analysis of Fig. 4 ▸ is added to the supporting information.

### 3.1. XRR and XRF analysis of TiO_*x*_/ML

**Figure 5:**
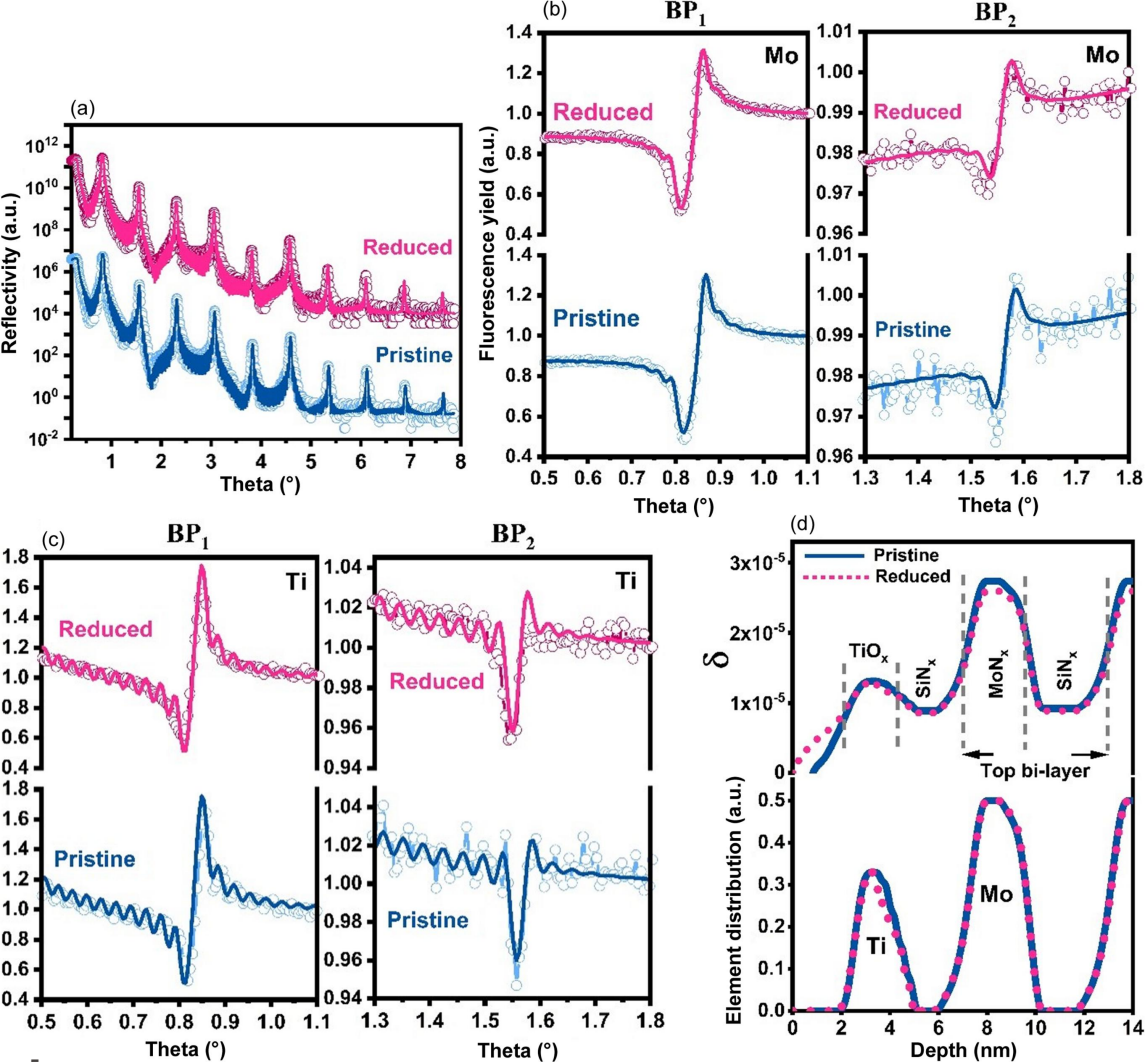
(*a*) XRR of the TiO_*x*_/ML sample in the pristine and reduced states. For clarity, reflectivity curves have been displaced vertically with respect to each other. The continuous solid line represents the best fitting to the experimental data. (*b*) XRF of Mo around BP_1_ and BP_2_ before and after reduction. (*c*) XRF of Ti measured around BP_1_ and BP_2_ before and after reduction. (*d*) Elemental distribution (for Ti and Mo) and the δ profile (showing only the top bilayer period of the multilayer) of the TiO_*x*_/ML sample in the pristine and reduced states. The continuous solid line and dotted line represent the pristine and reduced states, respectively. No change in the total width of Ti element distribution was observed after reduction.

Fig. 5 ▸(*a*) shows the XRR curves of sample TiO_*x*_/ML in the pristine and reduced states. In the reflectivity curves, BPs up to the nineth order are clearly visible. Figs. 5 ▸(*b*) and 5 ▸(*c*) show Mo and Ti fluorescence yield, measured around the first and second BPs, for the TiO_*x*_/ML sample in the pristine and reduced states. Figs. 5 ▸(*a*)–5(*c*) show that the fitted reflectivity and fluorescence curves are in good agreement with the experimental data. The best-fit profiles are shown in Fig. 5 ▸(*d*) as the real part of the decrement of the optical constant δ and by the elemental distribution of Ti and Mo. The model of the ML assumes that all MoN_*x*_/SiN_*x*_ bilayers are identical. The best-fit bilayer thickness of the multilayer is 5.9 nm for both pristine and reduced samples. A slight decrease in δ values for MoN_*x*_, SiN_*x*_ and Ti after reduction was attributed to thermal annealing during the reduction process. The thicknesses of TiO_*x*_ and the capping SiN_*x*_ layer were calculated using the full width at half-maximum of their respective elemental distribution. The calculated thicknesses were 1.8 nm for TiO_*x*_ and 2.8 nm for SiN_*x*_. No change in the thickness of these layers was observed after reduction.

A small broadening at the TiO_*x*_-on-SiN_*x*_ interface was observed after reduction, as can be seen in the elemental distribution in Fig. 5 ▸(*d*). This could be due to thermal annealing during the reduction. To fit the data, a 1 nm-thick layer on the TiO_*x*_ surface was added to the fitting model. This layer was expressed as a gradient at the ambient or air/TiO_*x*_ interface. Such layers can be explained by the presence of adventitious carbon on the TiO_*x*_ surface, which was determined using XPS. After reduction, an increase in its thickness (∼0.9 nm) compared with the pristine state was observed as shown in the δ profile of Fig. 5 ▸(*d*). Details of the XPS measurements and results are added in the supporting information. Fig. 5 ▸(*d*) shows that the Ti distribution in TiO_*x*_ is not changed by the reduction of the sample without NPs, as already predicted by qualitative analysis.

### 3.2. XRR and XRF analysis of Co/TiO_*x*_/ML

**Figure 6:**
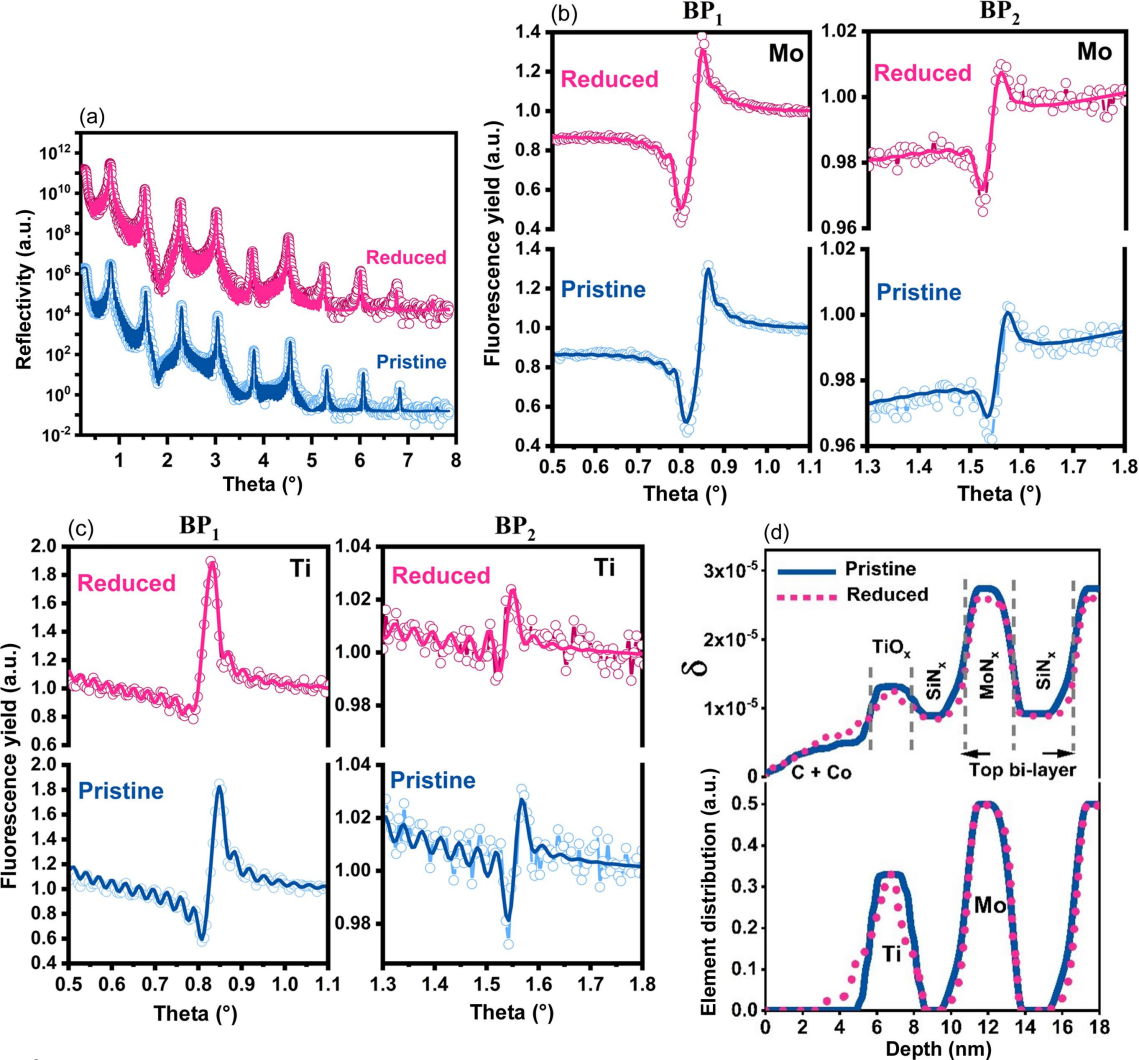
(*a*) XRR of the Co/TiO_*x*_/ML sample before and after reduction. For clarity, reflectivity curves have been displaced vertically with respect to each other. The continuous solid line represents the best fit to the experimental data. (*b*) XRF of Mo measured around BP_1_ and BP_2_ before and after reduction. (*c*) XRF of Ti measured around BP_1_ and BP_2_ before and after reduction. (*d*) Elemental distribution (for Ti and Mo) and δ profile (showing only the top bilayer of the multilayer) of the sample in the pristine and reduced states. The continuous solid line and dotted line represent the pristine and reduced states, respectively. A change in the total width of the Ti profile after reduction can be seen.

Fig. 6 ▸(*a*) shows the XRR curves of the Co/TiO_*x*_/ML sample in the pristine and reduced states. The representation of fits and results on Fig. 6 ▸ follows the same structure as for Fig. 5 ▸ for ease of comparison. As for the TiO_*x*_/ML sample, the fitted reflectivity and fluorescence curves of Co/TiO_*x*_/ML match well with the experimental data. A slight decrease in δ values for MoN_*x*_, SiN_*x*_ and Ti was observed after reduction, similarly to the TiO_*x*_/ML sample. A 2.4 nm thickness of the TiO_*x*_ layer in the pristine state was calculated in a similar manner as for the TiO_*x*_/ML sample. As already mentioned, the Co NP layer was modeled in the effective medium approach as a Co gradient layer. Here we assumed that Co atoms were mixed with a C layer on the outer surface, and with TiO_*x*_ at the Co-on-TiO_*x*_ interface. Such a model can describe any shape of Ti profile realistically but may not represent a realistic Co distribution. This is a limitation of the technique: analyzing the Co distribution would require a different ML design and was beyond the scope of this study.

In the qualitative analysis of the Ti fluorescence yield of sample Co/TiO_*x*_/ML, it was shown that after reduction at 600°C the phase of Ti fluorescence changed in comparison with its pristine state [Figs. 4 ▸(*c*) and 4 ▸(*d*)]. The change in the phase of Ti fluorescence indicates a change in the Ti atomic distribution at the Co/TiO_*x*_ interface or movement of Ti atoms in an upward direction with respect to the position of the ML period. Fig. 6 ▸ shows the results of the quantitative analysis of the Co/TiO_*x*_/ML sample before and after reduction. Figs. 6 ▸(*a*)–6 ▸(*c*) show that simultated XRR and XRF curves fit perfectly to the measured data. The best-fit model is shown in Fig. 6 ▸(*d*). By comparing the elemental distribution of Ti before and after reduction in Fig. 6 ▸(*d*), we note the presence of an ∼2.1 nm Ti-containing layer at the Co-on-TiO_*x*_ interface. This layer has a noticebly low Ti atomic concentration compared with the pristine TiO_*x*_ layer. We attribute such a shifted layer of Ti atoms over the Co NP layer to the encapsulation effect of SMSI. This is consistent with the TiO_*x*_ overlayer formation over Co NPs after the high-temperature reduction, which persisted after passivation with O_2_ at a low temperature under air exposure (O’Shea *et al.*, 2011 ▸).

## 4. Discussion

**Figure 7:**
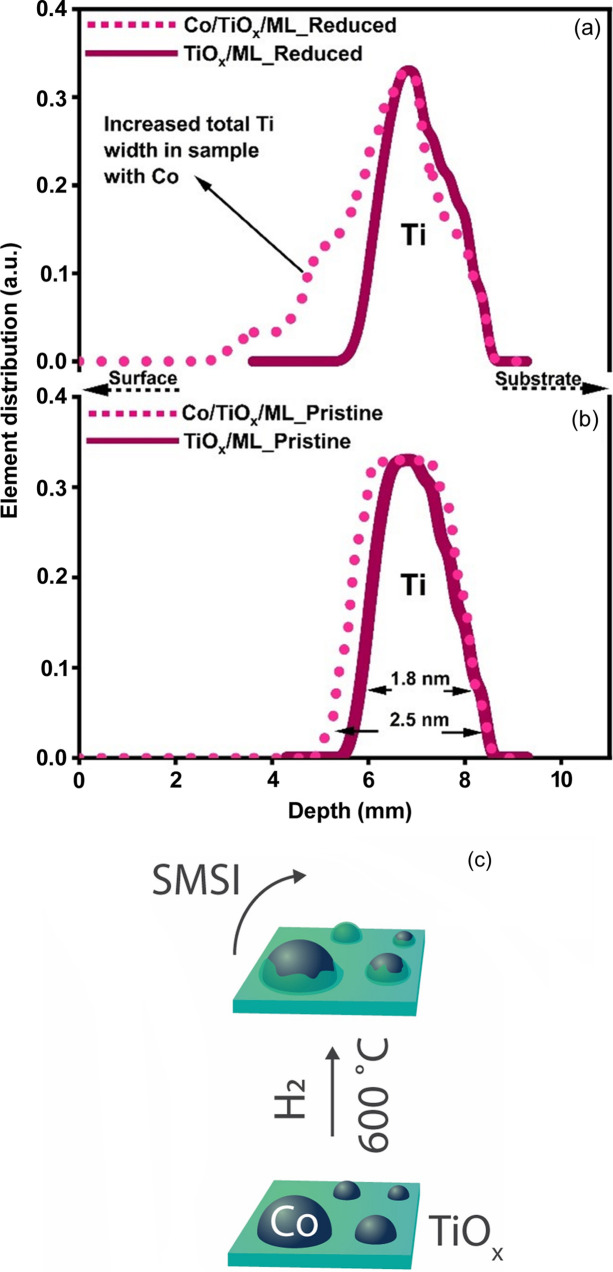
(*a*) and (*b*) Comparison of Ti elemental distribution profiles of TiO_*x*_/ML and Co/TiO_*x*_/ML samples in the (*a*) reduced and (*b*) pristine state. (*c*) Schematic representation of TiO_*x*_ migration on Co NPs upon high-temperature reduction.

The scope of this study was to give proof of concept that SMSI could be detected at the NP-ensemble level using the XSW technique. Figs. 7 ▸ (*a*) and 7 ▸(*b*) show a comparison of Ti elemental distributions in best-fit models for the TiO_*x*_/ML and Co/TiO_*x*_/ML samples in the reduced and pristine states, respectively. Here, we clearly observe that the shift of Ti atoms in the direction of the surface was only observed in the presence of Co NPs and after high-temperature reduction. The observed shift of about 2 nm is small compared with the height of the Co NPs revealed by AFM (>20 nm) and TEM (5 nm), suggesting only partial coverage of the NPs by a TiO_*x*_ layer at the moment of analysis, as also confirmed by TEM-EDS (Fig. 2 ▸). The observed partial coverage of Ti might be caused by different factors, such as Ti segregation on top of smaller nanoparticles only, for example, with diameters of ∼3–5 nm, or the removal of some of the Ti from the Co NPs due to Ti oxidation by exposure to air after reduction. Oxidation and (partial) removal of TiO_*x*_ overlayers were indeed observed for Ni/TiO_2_ (Monai *et al.*, 2023 ▸) and Au/TiO_2_ (Liu *et al.*, 2019 ▸) when changing from reducing to more oxidizing environments, such as CO_2_ hydrogenation conditions or 10% O_2_/He.

Note that, although the layered structure is a model system for catalysis, it is already closer to a real catalyst since we have shown that the SMSI can be characterized with variable-sized NPs. As such, this model can also be considered as the advance catalyst model. To obtain a more quantitative interpretation of XSW data, size-selected NPs should be used, which can be prepared for example by colloidal chemistry approaches (Puntes *et al.*, 2002 ▸) and by size-selected spark ablation. Various coverage and NP spatial distribution could be modeled by deconvoluting the information about changes of NP positioning assuming a fixed shape and size. It should be then possible to reconstruct not only the overlayer metal oxide profile but also the atoms from the metal NP layer. Moreover, for well defined NP sizes, one could analyze the redistribution of NPs before and after reduction from the differences in effective metal atomic profiles. The potential of such analysis should be further investigated.

To obtain more information about the details of SMSI, *in situ* and *operando* characterization experiments can be proposed. Since X-rays can penetrate through a gas environment, we plan to study the possibility of *operando* characterization. Currently, the biggest challenge in such an experiment will be the protection of the XRF detector from the reaction environment and efficient cooling, so preserving the minimum possible barriers between the sample and the detector to limit the absorption of XRF signal. Another challenge of using a laboratory instrument for such measurements could be the integration of the fluorescence detector with the instrument. Since both the measurements were done simultaneously, synchronization of the two measurements should be foreseen. The sealed tubes with fixed wavelength used in the laboratory setup also limit the selection of elements to those from which fluorescence can be excited; however, practically, these are minor limitations that can be easily overcome. On the other hand, the ease of implementation and minimal requirements of the XSW technique, such as a thermally stable multilayer, make it a robust and possibly routine analysis tool for investigating the encapsulation effect of SMSI. Outside the field of catalysis, we envision that the thermally stable periodic ML developed herein could be suitable for studying metal segregation and alloying after and during thermal treatment, relevant for metallurgy and nanotechnology.

## 5. Conclusions

In this study, we have demostrated a proof-of-concept application of XSWs to study the encapsulation of Co NPs by thin TiO_*x*_ layers due to SMSI. To achieve this, we developed an XSW generator, compatible with the requirements for SMSI study, namely a thermally stable periodic multilayer structure based on MoN_*x*_/SiN_*x*_ layers. On top of the ML, we deposited a thin film of TiO_*x*_ followed by Co NPs to prepare a flat model catalyst resembling the high-surface-area counterpart Co/TiO_2_ Fischer–Tropsch catalyst. Two samples, TiO_*x*_/ML (reference) and Co/TiO_*x*_/ML, were studied, after sample preparation and after reduction at 600°C for 1 h in H_2_/N_2_ to induce SMSI. We have shown that even qualitative analysis of angle-resolved XRF data immediately indicates a change in Ti atomic distribution, which can be a sign of SMSI. The rigorous free-form analysis of combined XRR and XSW data provided a quantitative interpretation of the changes in Ti atomic distribution induced by high-temperature reduction. Ti atoms diffused up to 2.1 nm into the Co NP layer, which indicated that only partial encapsulation of >20 nm Co NPs persisted after reduction, passivation and air exposure. Our results demonstrate the use of XSWs in the catalyst field. Future efforts should be dedicated to developing an *in situ* or *operando* XSW study to follow structural changes of surfaces under conditions relevant to catalysis and other thermal-activated processes.

## Supplementary Material

Supporting figures. DOI: 10.1107/S1600576724001730/ge5144sup1.pdf
