# Supplementary material for: X-ray standing wave characterization of the strong metal–support interaction in Co/TiO x model catalysts
Source: J Appl Crystallogr. 2024 Mar 31;57(Pt 2):481–91. doi: 10.1107/S1600576724001730 (PMC11001393; doi:10.1107/S1600576724001730)
Supplement: Supplementary file 1 [file j-57-00481-sup1.pdf]

JOURNAL OF
APPLIED
CRYSTALLOGRAPHY

Volume 57 (2024)

Supporting information for article:

X-ray standing wave characterization of the strong metal-support interaction in Co/TiOx model catalysts

Atul Tiwari, Matteo Monai, Ksenia Matveevskii, Sergey N. Yakunin, Laurens D. B. Mandemaker, Martina Tsvetanova, Melissa J. Goodwin, Marcelo D. Ackermann, Florian Meirer and Igor A. Makhotkin

S1. XPS analysis

Sample TiOx/ML was transferred from ambient environment into a Thermo-Fisher Theta probe Angle Resolved X-ray photoelectron spectrometer which uses monochromatic Al-Kα radiation (1486 eV) with a spot size of 400 µm. Measurements were carried out at a pressure in the order of 1×10^{-9} mbar as measured by an ion gauge. Angle resolved XPS spectra for C1s is reported with respect to the surface normal.

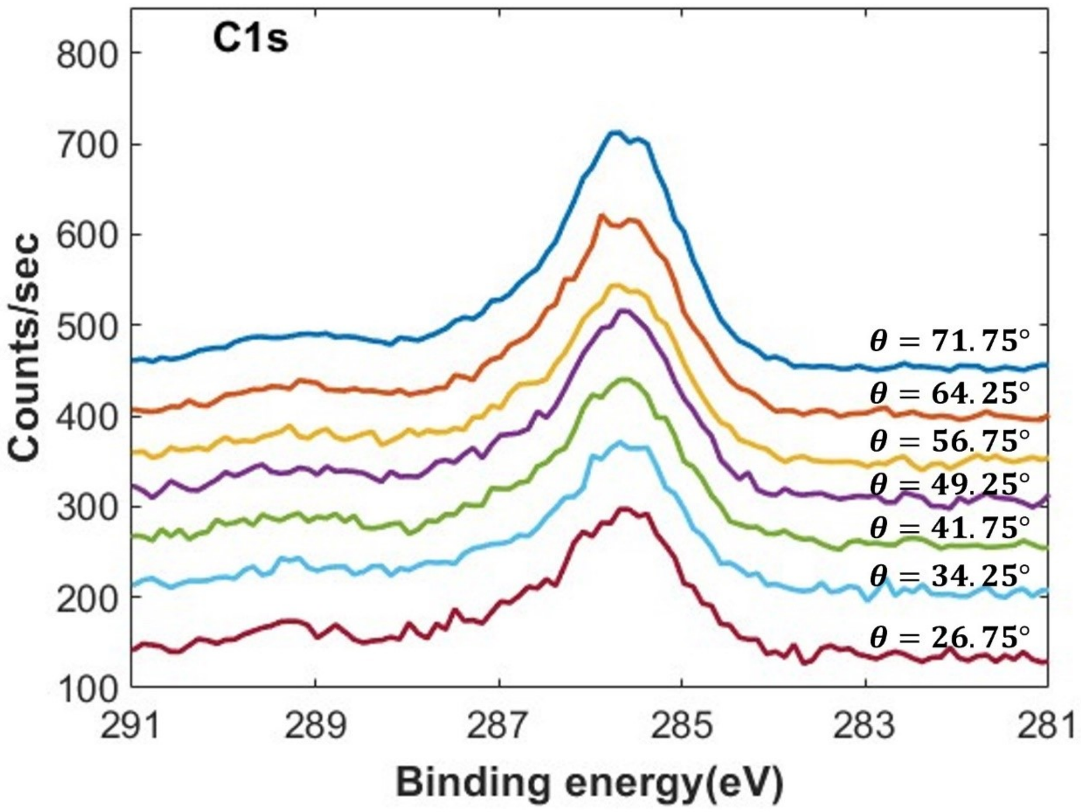

Figure S1 Angle resolved XPS spectra of sample TiO_x/ML in the pristine state showing the presence of adventitious carbon on TiO_x surface.

S2. Annealing test of the ML at 600 °C for 1h

The ML sample was annealed at 600 °C for 1 hour, twice in a vacuum environment. As seen from the graph, no shift in the Bragg reflection can be observed after annealing, which could indicate a change in the bilayer period of the ML. This shows that the ML structure is thermally stable at 600 °C, which is necessary during the reduction process.

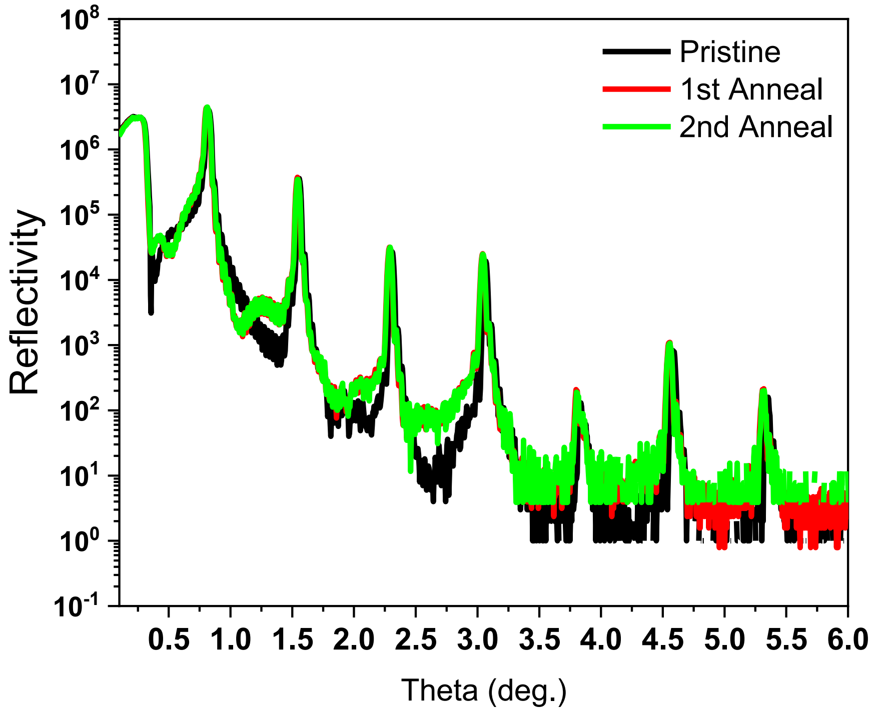

Figure S2 X-ray reflectivity of MoN_x/SiN_x sample in the pristine and after 1st and 2nd annealing at 600 °C for 1h.

S3. Additional AFM images of sample Co/TiO$_x$/ML

Below are additional AFM images of the samples prepared in this study.

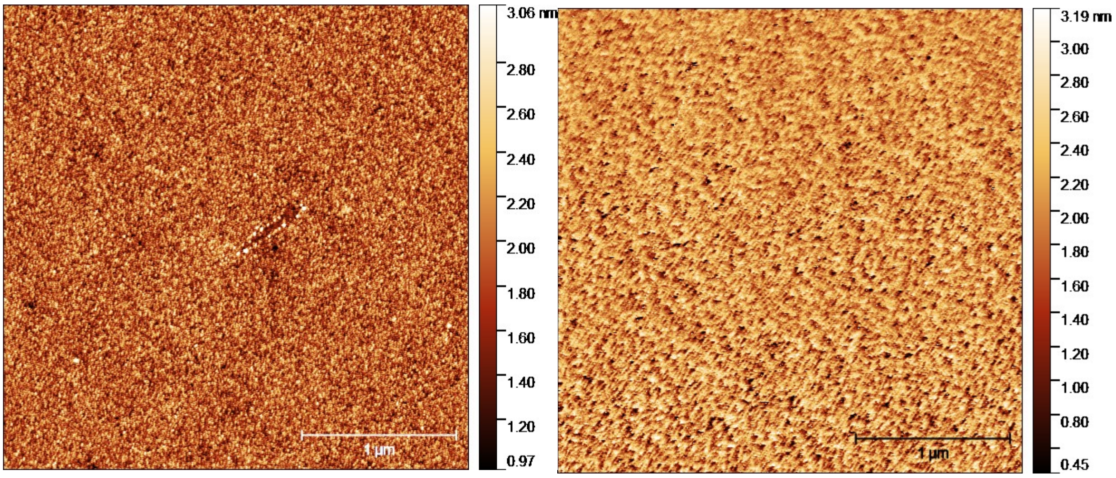

Figure S3 AFM images of TiOx/ML after reduction at 600 °C for 1h. Surface roughness = 0.33 nm (left) - 0.42 nm (right). Median height value =1.92 nm (left) – 2.07 nm (right)

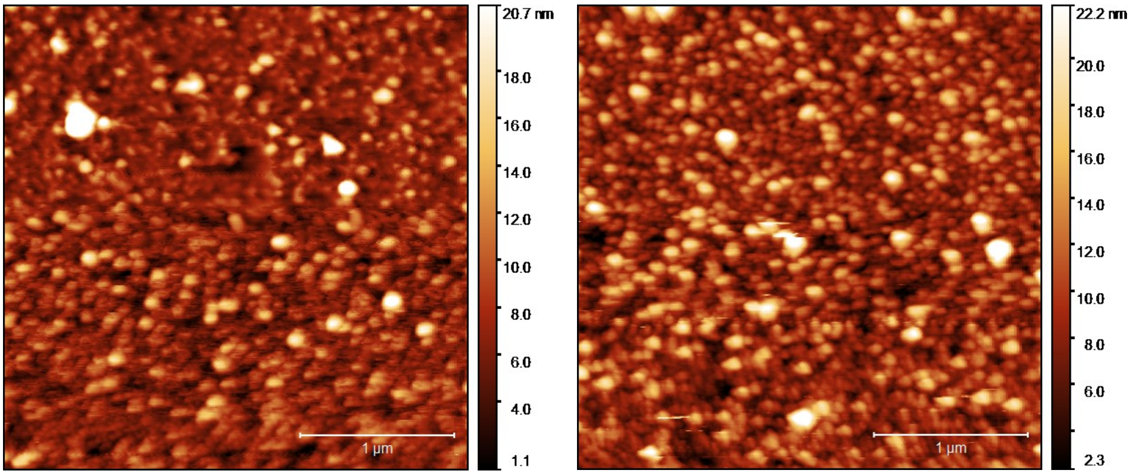

Figure S4 Additional AFM images of Co/TiOx/ML after reduction at 250 °C for 1h. Surface roughness = 3.01 nm (left), 3.07 nm (right).

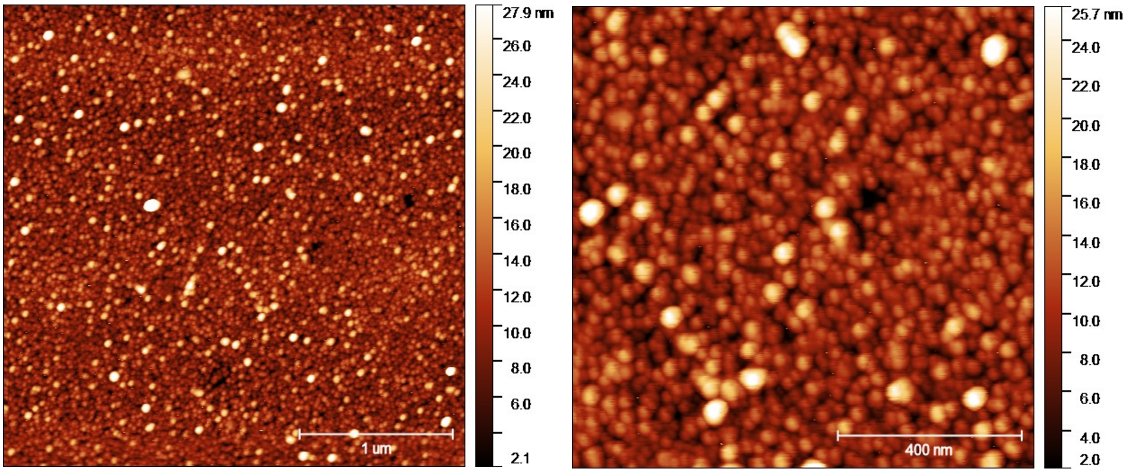

Figure S5 Additional AFM images of Co/TiOx/ML after reduction at 600 °C for 1h. The right image is a zoom-in of the left image. Surface roughness = 3.50 nm (left), 3.39 nm (right)

S4. Detailed explanation of Figure 4

Figure 4(a) & (b) gives the Ti fluorescence yield of sample TiO_x/ML, measured around BP_1 and BP_2 respectively in their pristine and reduced state. In the pristine state as shown in figure 4(a), a decrease followed by rise in the Ti fluorescence suggesting that the overlap of TiO_x layer at BP_1 is more on anti-node than node. Similarly, in Figure 4(b), sharp decrease in the Ti fluorescence suggests that at BP_2 the position of TiO_x layer is centered at/near the node. This correspond to the shift of the mean atomic distribution by the ~1/4 of the XSW period corresponding to ~1.5 nm. After reduction of sample TiO_x/ML, no major changes in the phase of Ti fluorescence measured around BP_1 and BP_2 are observed in compare to pristine state. A slight angular shift can be attributed to small changes in the bi-layer period of the ML structure after annealing that was part of the reduction process. It indicates that in sample TiO_x/ML, even after high temperature reduction, the Ti atomic distribution profile at the surface is similar to the pristine state. It corresponds to no movement of Ti atoms at the surface after reduction.

Figure 4(c) & (d) gives the Ti fluorescence yield of sample Co/TiO_x/ML, measured around BP_1 and BP_2 respectively in their pristine and reduced state. In pristine state, in Figure 4(c) a decrease followed by rise in the Ti fluorescence indicates that at BP_1, the overlap of the TiO_x layer is more on the anti-node than node. In Figure 4(d) for pristine state, a decrease followed by rise in the Ti fluorescence of approximately similar amplitude reflects that at BP_2, the overlap of TiO_x layer is approximately equal on both node and anti-node. After reduction, the fluorescence yield of Ti shown in Figure 4(c) and 4(d) shows that the overlap of TiO_x layer on the node and anti-node is changed in compare to its pristine state. Around BP_1, the overlap of TiO_x layer is increased on the anti-node, resulting a small decrease followed by a sharp rise in the Ti fluorescence. Around BP_2, the overlap of TiO_x layer on the node and anti-node is unequal. It is more on the anti-node than node resulting a small decrease followed by a rise

in the Ti fluorescence. The changes in the overlap of Co/TiO_x interface on the node-and anti-node in compare to its pristine state indicates that, after reduction migration of Ti atoms to the Co NPs surface is taking place. This migration of Ti atoms was not visible in the sample TiO_x/ML.

S5. Error corridors in delta profile and element distribution and correlation matrix for sample Co/TiO_x/ML

Figure S6 & S7 show the error corridors in δ profile and element distribution for the sample Co/TiOx/ML after reduction at 250 °C and 600 °C. The narrow error corridors shows confidence in the validity of the best-fitting model. The absence of a chessboard pattern in the correlation matrix eliminates the possibility of an over-defined fitting model from consideration.

(a) **(b)**

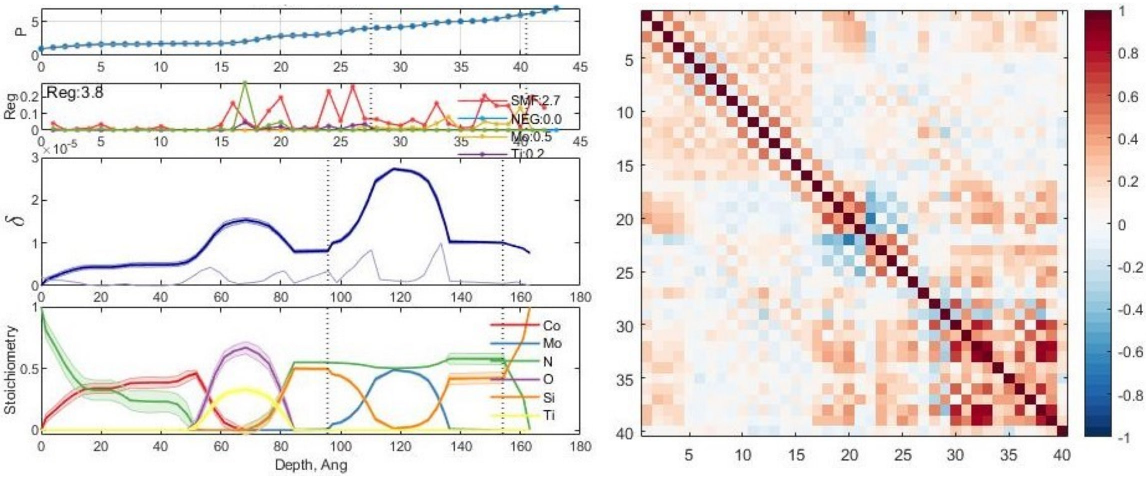

Figure S6 (a) Error corridors in δ profile and element distribution, (b) correlation matrix for sample Co/TiOx/ML after reduction at 250 °C.

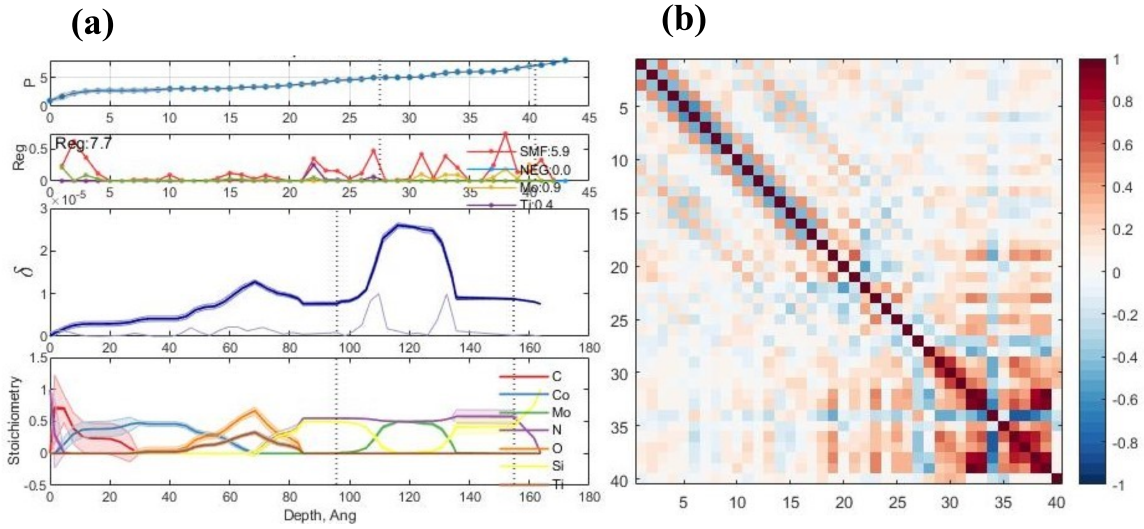

Figure S7 (a) Error corridors in optical constant δ profile and element distribution, (b) correlation matrix for sample Co/TiO_x/ML after reduction at 600 °C.

S6. Element distribution of Si, Ti and Mo for the sample TiO_x/ML and Co/TiO_x/ML in their pristine state as well as after reduction.

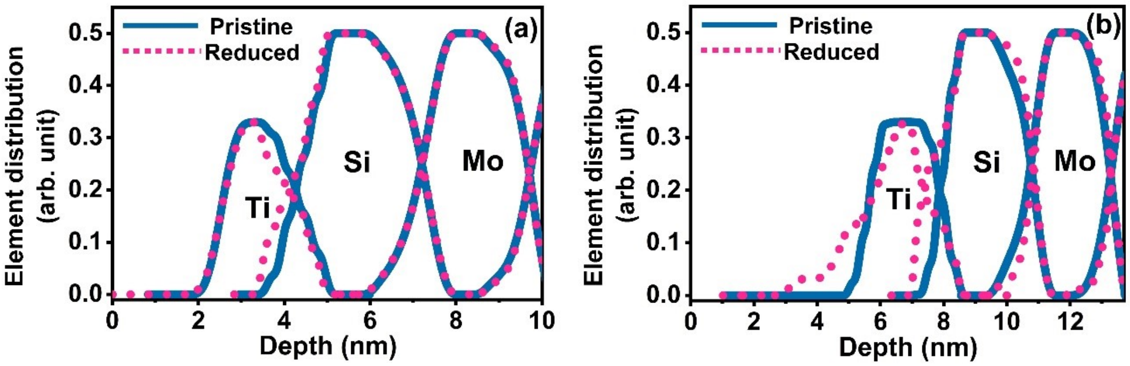

Figure S8 (a) Element distribution of Si, Ti and Mo for the sample TiO_x/ML in pristine state and after reduction (b) Element distribution of Si, Ti and Mo for the sample Co/TiO_x/ML in pristine state and after reduction.
